# Menorraghia's impact on quality of life: A case control study from a teaching Hospital in Lahore

**DOI:** 10.12669/pjms.346.15410

**Published:** 2018

**Authors:** Lamia Yusuf

**Affiliations:** 1 *Dr. Lamia Yusuf, MBBS, FCPS, MHPE. Assistant Professor (Gynaecology / Obstetrics), Rashid Latif Medical College/ Arif Memorial teaching Hospital, Lahore, Pakistany*

**Keywords:** Menorrhagia, Quality of life, Treatment

## Abstract

**Background and Objective:**

Menorrhagia is one of the common complaints presenting in gynaecology outdoor and clinics. Menorrhagia is defined as excessive uterine bleeding occurring at regular intervals or prolonged uterine bleeding lasting for more than seven days. This is a very distressing condition effecting almost all ages, and has multiple causes. The objective of this study was to determine effect of menorrhagia on quality of life of a woman.

**Methods:**

It was a case control study, conducted at Arif Memorial Teaching Hospital Lahore from January 2017 to December 2017. It included 230 women. Out of these, 150 women who had menorrhagia were included in Group A (cases). Group B included 80 women accompanying them and have normal menstrual cycles (controls).

**Results:**

The mean age of the participants was 35.56±8.85 years. Duration of menorrhagia was more than 4 years in 11% of the patients, 22% had menorrhagia for 1-2 years and 1.2% for three months. Among all age groups, quality of life was better in female without menorrhagia as compared to female with menorrhagia (p-value <0.001).

**Conclusion:**

Health care providers should have knowledge to treat patients with menorrhagia, also future qualitative studies should be done to determine perception of patients regarding treatment and management of menorrhagia.

## INTRODUCTION

Menorrhagia, although is not life threating, but it has a major impact on personal, family and social life of a woman, effecting her household work and workplace responsibilities. It is defined as menstruation periods at regular cycles but with excessive flow which may last for more than seven days. Menorrhagia can cause menstrual bleeding of more than 80 mL in each cycle.^1^,^2^

In treating menorrhagia the primary aim should be to improve quality of life of woman. WHO defines Quality of Life as individuals’ perception of their position in life in the context of the culture and value systems in which they live and in relation to their goals, expectations, standards and concerns. It is a complex phenomenon.^3^ One in five women end up in hysterectomy by the age of 60 years^1^ and most of them have no detectable pelvic pathology. The high cost associated with medical management, means that menorrhagia places a huge financial burden on the family.^1^

The overall aim of management of menorrhagia is to reduce the adverse impact of the condition thereby improving the sufferer’s ‘quality of life’ (QoL). Management of menorrhagia depends upon the threshold of patient and her expectations about her symptoms. Exact amount of blood loss is almost difficult to measure. Assessment of menstrual flow is highly subjective and gauging the severity of the condition by objective assessment of menstrual blood loss is impractical, the use of QoL instruments in menorrhagia research is increasing to allow more objective assessment of clinical outcomes of therapeutic interventions. The objective of this study was to determine effect of menorrhagia on quality of life of a woman.

## METHODS

A case control study was designed, 360 women who presented in Outpatient Gynaecology & Obstetrics Department of AMTH (Arif Memorial Teaching Hospital Lahore) from January 2017 to December 2017 (1 year) were included in study through purposive sampling. Sample size was calculated using open epi tool. Those patients who had menorrhagia were included in group A (cases; n=160). Group B comprises of Age group matched controls. The participants were interviewed by using a questionnaire and SF 36 questionnaire of quality of life. The questionnaire was prepared by author through literature review, the questions were based on history, symptoms and severity of diseases. The severity of menstural symptoms and sign were determined from answers to questions about duration of periods, number of pads and additional clothes, tampons used, experience of flooding and passing clots, frequency of blood staining of clothes and extent of dysmenorrhea.

**Table-I:** Comparison of quality of life in female with or without menorrhagia controlling for age.

Age Group	Menorrhagia	Mean	Std. Deviation	p-value
<20	Yes	17.14	1.57	<0.001*
	No	6.50	2.78	
21-25	Yes	13.38	3.88	<0.001*
	No	4.00	2.07	
26-30	Yes	15.13	3.38	<0.001*
	No	4.49	2.55	
31-35	Yes	15.18	2.78	<0.001*
	No	5.17	3.49	
36-40	Yes	16.37	2.54	<0.001*
	No	10.50	0.58	
>40	Yes	16.73	2.57	<0.001*
	No	3.00	0.00	

* Independent sample t test, P-value significant at 0.001

The San Francisco-36 Health Survey is a multi-purpose, short-form health survey which contains 36 questions. It yields an eight-scale profile of scores as well as summary physical and mental measures. It measures health-related quality of life on eight dimensions which include physical functioning, social functioning, role limitations due to physical problems, role limitations due to emotional problems, general mental health, energy and vitality, bodily pain and general health perceptions.^4^

This is a broad instrument and it has been validated for menorraghia in a study by Sculpher MJ et al.^5^, but it has certain limitations, that are discussed later. We obtained ethical approval from ethical review board of the institution. Participants were asked to give consent. Data was entered into SPSS 21 and results were obtained as frequency, mean and p values. Mean and percentage were calculated for SF36 questionaire and response to questions was presented in the form of frequency and percentage. Independent sample T test was used to determine the statistically significant difference. P-value of 0.05 or less was considered as significant.

## RESULTS

In this study 230 females were included. The mean age of the participants was 35.56±8.85 years. Regarding duration of menorrhagia; 11% of the patients had menorrhagia for more than four years, 22% had menorrhagia for 1-2 years and 1.2% for three months. Responding to the question about duration of bleeding during each cycle; 16% had bleeding for three days, 70% had bleeding for 4-8 days and 11.9% had bleeding for more then 8-12 days. Among those who had menorrhagia, 33.0% used 2-3 pads per day, 47.2% 4-6 pads/day, 15.1% 6-8 pads/day, 4.1% 8-10 pads per day and 6% used more than 10 pads per day; thus contributing to huge financial burden on the family.

**Graph. 1:**
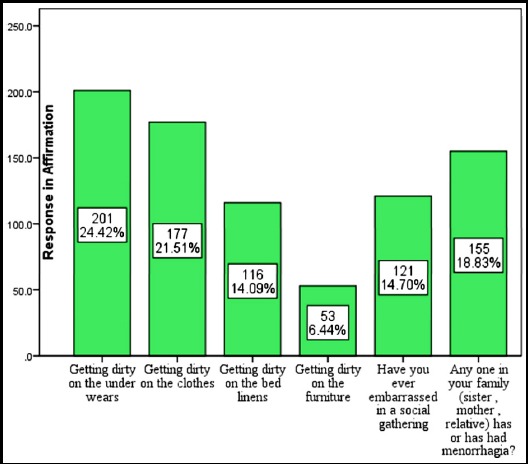
Experiences of patient in different situations in menorraghia.

We compared quality of life in both case and control groups and the results showed that among all age groups the quality of life was better in female without menorrhagia as compared to those with menorrhagia (p-value <0.001).

## DISCUSSION

This study was conducted to determine the magnitude of menorrhagia on the quality of life of a woman. Review of different studies shows that although menorraghia is non-life threatening, condition, but it has a tremendous effect on quality of life of female. It also has a negative effect on their family life.^6^ WHO defines health as “A state of complete physical, mental, and social well-being not merely the absence of disease” (measuring quality of life). In recent days the perception of health has been broadened and now it includes social, psychological and physical aspect of a life.^7^. The other development is shifting of quality of life from physician’s perception to the individual ‘s subjective feeling of wellbeing.^8^ Sf 36 is a generic instrument and used in this study to see all above described aspects on QOL of a patient suffering from menorraghia. But the analysis of the disease related effects has been limited by available tools for measuring outcome of the disease process and effect on quality of life. That is why it is necessary to develop health related quality of life instruments regarding menorraghia or any other abnormal bleeding, so objective assessment of quality of life can be carried out. This study is an attempt to see the impact of menorrhagia on quality of life of a woman.

High cost related to treatment of menorrhagia, adds a huge financial burden on the family, the estimated annual direct and indirect financial burden is actually under estimated. In a systemic review conducted by DeaN BB etal the annual cost of treatment of menorraghia/abnormal uterine bleeding in USA was $1 billion The result found in my study disclosed that cost in terms of rise in number of pads used for protection was bigger in case group. Still future studies are required to estimate annual cost on treatment and management of menorrhagia particularly in Pakistan.^9^ In poor countries like Pakistan where day to day needs of a family are meet with great struggle, women usually ignore such problems as emphasized by Hussain et al 10 The social taboo associated with speaking about periods force women to stay silent thus resulting in medical problems, like anemia., and psychological stress. These facts hamper the physical and mental well-being of a woman, who actually act as a back bone of her family thus effecting HEALTH of whole family.

Clinical findings that are most strongly related with blood loss volume are the rate of changing sanitary protection during full flow, the total number of pads used, iron status of a woman, the size of clots and need to change protection during social gatherings or during nights. In routine clinical practice we usually depend on on these subjective findings based on perception of a woman, but there is no reliable tool that can assess blood loss accurately. It is apparent that all these symptoms and sign were increased in case group. In our study women describe severe symptoms after menorraghia hence they used more than one product together for protection, they experience soaking of their clothes and bed sheets. Likewise, they experience more pain. These findings were comparable to the findings discussed by Gokyildiz S et al.^1^ and Warner PE et al.^12^ There are many possible cause of menorraghia like fibroids, endometriosis, adenomyosis, bleeding disorders, endometrial hyperplasia, and many more, but objective of treatment of all causes is twofold, first is to reduce or stop menorraghia and second is to improve quality of life.^13^,^14^

In our study the quality of life was better among all age groups in control group (without menorrhagia) as compared to case group (with menorrhagia) p-value <0.001. Alike findings were observed in literature that menorraghia disturb quality of life in an undesirable way.^13^ According to one more study by Kadir RA et al the health-related quality measured by SF36 was below 25^th^ percentile for the US general female population suffering from menorraghia.^15^

### Limitations of the study

A validated Urdu version of SF36 questionnaire was lacking. Sf 36 is inappropriate to be used because outcome of measure taken in treatment of menorraghia are not addressed. So, it is recommended that specific disease related tool that measure quality of life in patients can be used for future study purpose. Further future research should be focused on qualitative aspect of this study so as to have in depth understanding about patient’s perception and experience with menorraghia. It will be better for effective ness of treatment and care given to the patients.

In conclusion doctors should have adequate knowledge and skill to treat menorraghia. Every woman should be evaluated individually and integrated care should be provided keeping in mind the physical, social, and psychological impact of this disease on women’s health.
